# Outcomes of Susanna UF implants in refractory congenital glaucoma

**DOI:** 10.1016/j.clinsp.2025.100619

**Published:** 2025-05-01

**Authors:** Aline Domingos Pinto Ruppert, Nara Gravina Ogata, Leopoldo Ernesto Oiticica Barbosa, Paulo Silas Neroni Stina, Marcus Vinícius Takatsu, Ernst Werner Oltrogge, Marcelo Hatanaka

**Affiliations:** Hospital das Clínicas da Faculdade de Medicina da Universidade de São Paulo (USP), São Paulo, SP, Brazil

**Keywords:** Primary congenital glaucoma, Glaucoma drainage device, Intraocular Pressure

## Abstract

**Objective:**

This study aimed to evaluate the success and complication rates of the Susanna UF Glaucoma Drainage Device (SUFGDD) in treating refractory primary congenital glaucoma.

**Patients and methods:**

In this prospective non-comparative clinical trial, patients with refractory primary congenital glaucoma, under 18-years-old, who had unsuccessful prior glaucoma surgeries, underwent standardized SUFGDD implant surgery. All procedures were performed by a single surgeon (A.D.P.R.). Pre and postoperative intraocular pressure (IOP), number of antiglaucoma eye drops, surgical complications, and any subsequent related events were recorded. Success criteria were defined as follows: (I) IOP ≥ 5 and ≤ 21 mmHg; (II) IOP ≥ 5 and ≤ 18 mmHg. Each group was further categorized as absolute success (without medication) or qualified success (including those with medication).

**Results:**

The study included 45 eyes from 31 patients, with an average age of 6.14 ± 5.02 years (range: 9 months‒18 years). The mean follow-up time was 37.78 ± 10.44 months. Mean IOP decreased from 28.18 ± 5.69 mmHg to 14.73 ± 2.90 mmHg (*p* < 0.05). The average number of antiglaucoma medications was reduced from 2.96 ± 0.74 to 1.40 ± 1.23 (*p* < 0.05). At 36-months postoperatively, the qualified success rates for criteria I and II were 93.3 % and 77.8 %, respectively. Seven eyes (15.6 %) experienced complications, all of which required reoperation; six cases were successfully managed, while one case resulted in permanent vision loss due to endophthalmitis. There were no significant associations between complications and analyzed risk factors (e.g., number of previous surgeries, age at surgery, past glaucoma surgeries, preoperative IOP, and number of preoperative eye drops). However, an association was observed between the occurrence of complications and surgical failure in the qualified success group for criterion II (HR = 6.50; 95 % CI 1.20‒31.35; *p* = 0.029).

**Conclusion:**

In this studied cohort, SUFGDD achieved qualified success rates of 93.3 % and 77.8 %, for criteria I and II, respectively, at 36-months, with a complication rate of 15.6 %.

## Highlights

• Primary congenital glaucoma is a leading cause of childhood blindness.
• Eyes with primary congenital glaucoma often require multiple surgeries to control pressure.
• Pediatric glaucoma drainage device studies show declining surgical success rates by year 3 due to complications.
• The Susanna UF Glaucoma Drainage Device has a published complication rate under 6 % in adults.
• No published data is available on the Susanna UF Glaucoma Drainage Device in pediatrics.

## Introduction

Primary Congenital Glaucoma (PCG) is a leading cause of childhood blindness, resulting in severe vision loss due to factors such as amblyopia, corneal scarring, refractive errors and optic nerve damage. Surgical intervention is critical and challenging, given the disease's aggressive and refractory nature.^1^, ^2^, ^3^

Though angle surgeries are generally the initial treatment choice, many eyes – particularly those with advanced disease – require additional procedures to achieve Intraocular Pressure (IOP) control.^1^, ^2^, ^3^, ^4^, ^5^, ^6^

Considering the severe long-term complications of antifibrotic drugs such as mitomycin C in trabeculectomies, along with the extended life expectancy of pediatric patients, Glaucoma Drainage Devices (GDDs) have been widely used in this population.^7^, ^8^, ^9^, ^10^, ^11^, ^12^, ^13^

Studies of GDDs, such as the Baerveldt® (Abbott Medical Optics, USA) and the Ahmed® models (New World Medical, USA), in pediatric cases, have demonstrated initial success rates between 80 %‒90.6 % within the first year; however, rates often drop to 40 %‒50 % by the third year,^14^, ^15^, ^16^, ^17^, ^18^, ^19^ primarily due to surgical failure and a high incidence of complications, including hypotony, cataract formation, retinal detachment, and choroidal effusion.^10^^,^^12^^,^^13^^,^^17^^,^^18^^,^^20^^,^^21^

Despite promising advances in GDD technology, many reports are limited by their retrospective nature, heterogeneity of glaucoma types, and short follow-up periods.^14^^,^^17^^,^^18^

Since becoming commercially available in Brazil in 2017, the Susanna UF Glaucoma Drainage Device (SUFGDD) (Kinner Silicone Rubber Indústria Comércio Ltda., Brazil) has shown a success rate exceeding 70 % and a complication rate under 6 %, with complications being generally mild and not associated with significant vision loss in adult studies.^22^^,^^23^ To our knowledge, there is no published data on SUFGDD in pediatric patients. Therefore, this study aimed to assess the long-term safety and effectiveness of the SUFGDD in children with primary congenital glaucoma.

## Objectives

1. To evaluate the success rates of SUFGDD in PCG.
2. To document complications associated with the use of SUFGDD in PCG.
3. To identify factors associated with complications and failure of SUFGDD in PCG.

## Materials and methods

This prospective, non-comparative clinical trial was conducted at the Congenital Glaucoma Clinic, Department of Ophthalmology, Hospital das Clínicas of the USP Medical School (HCFMUSP), Brazil, and adheres to standardized TREND (Transparent Reporting of Evaluations with Nonrandomized Designs) guidelines. This study was approved by the HCFMUSP Research Ethics Committee (CAAE: 37,138,920.5.0000.0068). Informed Consent Form was signed by the legal guardians of all participants.

The inclusion criteria were:

• Primary congenital glaucoma diagnosed before the age of 3-years.
• Patients aged 0 to 18-years.
• Refractory to previous antiglaucoma surgeries.
• Poor Intraocular Pressure (IOP) control.

Primary congenital glaucoma was defined by increased corneal diameter and Haab's striae as mandatory criteria, with one or more additional features: IOP > 21 mmHg, optic nerve cupping, or increased axial length. Poor IOP control was defined as IOP > 21 mmHg despite previous surgeries and topical treatment.^22^^,^^24^^,^^25^

The corneal diameter was considered increased when it exceeded 11 mm in newborns, 12 mm in children under one year of age and 13 mm at any age.^3^

Exclusion criteria included:

• Age over 18-years.
• Other types of glaucoma (secondary, juvenile, etc.).
• Presence of associated ocular anomalies identified during anamnesis or clinical examination (e.g., tumors, trauma, malformations, uveitis, chronic use of corticosteroids).
• Associated systemic anomalies.
• Previous non-antiglaucoma eye surgeries (e.g., vitrectomies, cataract surgeries).
• Previous glaucoma drainage implant surgeries.
• Inability to undergo ophthalmologic examination, even under anesthesia, due to social or clinical conditions during the follow-up period.
• Ocular conditions preventing adequate IOP measurement.

### Methodology

Patients who met both inclusion and exclusion criteria were invited to participate and subsequently underwent SUFGDD surgery between January 2018 and March 2020, with follow-up extending to December 2022. At the qualifying visit, data collection included age, gender, number of previous surgeries, corneal diameter, applanation tonometry, eye drop use, and cup-to-disc ratio.

At baseline, all consenting patients underwent assessment of Goldmann applanation tonometry, slit-lamp biomicroscopy, gonioscopy using the Zeiss 4-mirror lens, and fundoscopic examination (when feasible). Snellen best-corrected visual acuity was measured in cooperative children, most commonly from three years of age.

Intraocular pressure was assessed by the same trained examiners using a calibrated Goldmann applanation tonometer or a Perkins tonometer, and optic nerve examination was performed using indirect ophthalmoscopy. In eyes with poor corneal transparency, complementary ultrasound was performed. Non-cooperative patients underwent examinations under sevoflurane anesthesia; sedation was unnecessary for cooperative patients.

The final postoperative IOP was defined as the last IOP recorded at the end of each follow-up period.

All the patients received the Susanna UF® Drainage Device.

### Device description

The SUFGDD is a non-valved silicone implant with a plate featuring two 4.0 × 1.0 mm extensions for scleral fixation. The anterior portion is secured with two double sutures 6 mm from the limbus, positioning the plate 10 mm from the limbus. The plate contains fenestrations, which allow fibrosis to transfix the plate and make it more fixed and less susceptible to micro-movements, enhancing implant stability. These fenestrations are also present in other implants, such as Ahmed® and Baerveldt® devices. The SUFGDD plate covers an area of 200 mm^2^ and is composed of soft silicone, allowing size adjustments for specific situations (e.g., small eyes). The device's thickness is 0.5 mm, compared to Ahmed's (1.9 mm) and Baerveldt's (0.84 mm). The tube has an internal diameter of 230 μm and an external diameter of 530 μm.

### Surgical technique

All surgeries were performed by the same experienced surgeon (A.D.P.R.), following a standardized technique. Procedures were conducted under general and subconjunctival anesthesia. A conjunctival incision was made posterior to and parallel with the corneal limbus, extending for 5 to 6 h, preferably in the superior temporal region.

A careful dissection was done along 10 mm anteroposteriorly in the subtenon plane between the horizontal and vertical rectus muscles, avoiding muscle isolation with hooks.

The tube was inspected and irrigated with saline via an insulin needle to confirm patency. The plate was positioned 9 to 10 mm posterior to the corneal limbus, with the feet fixed to the sclera using 6‒0 silk double sutures, 6 mm from the limbus. The silicone tube was beveled to extend 2 to 3 mm into the anterior chamber, with the bevel oriented anteriorly at approximately 30°. A 6‒0 polyglactin suture was placed near the tube-plate junction to achieve complete lumen occlusion, verified by the inability to irrigate with balanced salt solution. The knot was leaved at the back of the tube (scleral face), to facilitate laser lysis of the suture, if necessary. Two fenestrations were created before ligation with a 6‒0 polyglactin needle, and a 26.5 G needle was used to guide the tube into the anterior chamber via a 2 mm scleral tunnel. The tube was placed anterior to the iris, parallel to it, and well posterior to the cornea. If deemed necessary, a third fenestration was created. The anterior tube segment was covered with a human donor scleral patch (8.0 × 6.0 mm), sutured to the episclera with 10‒0 nylon.. The conjunctiva and the Tenon capsule were closed with 6-0 polyglactin sutures.

The eye was checked for leaks as the anterior chamber was inflated to appropriate pressure using balanced salt solution.

### Postsurgical assessments

Postoperative care included topical corticosteroid (prednisolone) every 2 h and antibiotic (moxifloxacin) four times daily for the first week. Topical atropine 1 % was applied twice daily for two weeks, and prednisolone was gradually tapered over eight weeks. Due to advanced optic nerve damage in this cohort, and to improve bleb quality, all eyes received topical glaucoma medication from the first postoperative day to prevent IOP spikes. Medications were added based on IOP in this sequence: dorzolamide hydrochloride, timolol maleate, prostaglandin inhibitor, and brimonidine tartrate (in patients > 20 kg).

Follow-up visits occurred on postoperative day-1, week-1, month-1, month-3, month-6, month-9, and month-12, and continued every six months thereafter.

Postoperative IOP, number of antiglaucoma medications, surgical complications, and any subsequent related events were recorded, in each visit, by four glaucoma specialists (ADPR, NGO, LEOB and PSNS), trained in a standardized manner for the study.

### Statistical analysis

Each eye (right or left) was considered a unit of measurement in analyses, through the use of biostatistical methods designed to deal with bilateral correlated data (generalized linear mixed effects model).

Data were analyzed using STATA 14.0 (StataCorp LP, College Station, TX, USA). Demographic and clinical data were summarized using descriptive statistics.Averages of preoperative and postoperative IOP and antiglaucoma medications were calculated. Kaplan-Meier survival analysis was performed to evaluate success rates over time, using different success criteria at specific postoperative intervals.

Success was defined by two criteria based on IOP values: (I) IOP ≥ 5 and ≤ 21 mmHg; (II) IOP ≥ 5 and ≤ 18 mmHg, further classified as absolute success (without medication) or qualified success (with medication), based on prior studies of glaucoma devices.^22^^,^^24^^,^^25^

Failure was defined as IOP above the upper or below the lower limit at the last follow-up visit. Survival curves between groups were compared using the Log-rank test, and Cox Proportional Hazard models were used to analyze factors associated with failure risk. Significance was set at *p* < 0.05. Outcomes analyzed included survival, implant failure, and postoperative complications. Complications or additional surgeries did not count as failure unless they led to blindness or failed to meet IOP success criteria. Firth logistic regression was applied to identify factors linked to complications.

## Results

From the initial sample of 35 children (49 eyes) who underwent surgery, 4-eyes from 4 children were excluded for the following reasons: two due to follow-up loss, one non-compliant with postoperative eye drop protocols, and one with Pierre Robin syndrome (secondary glaucoma). Consequently, the final sample included 45 eyes from 31 children who received the Susanna UF® implant. Sixteen children (51.61 %) were male. The average age at implantation was 6.14±5.02 years (range, 9 months–18 years) (Table 1).

**Table 1:** Summary of the main variables relating to the study participants.

**Age (years) at SUFGDD implantation**	6.14 ± 5.02 (0.75‒18) (median 4.58)
**Gender – n (%)**	
Male	16 (51.61)
Female	15 (48.39)
**Glaucoma – n (%)**	
Bilateral	27 (87.09)
Unilateral	4 (12.90)
**Laterality eye, n (%)**	
Right	20 (44.44)
Left	25 (55.56)
**Corneal Diameter**	12.84±0.75 mm (median 12.90)
**Number of previous surgeries**	3.62±1.43 (median 3)

All children had Primary Congenital Glaucoma (PCG), with 4 cases (12.90 %) unilateral and 27 (87.09 %) bilateral. The average corneal diameter at surgery was 12.84±0.75 mm. Visual acuity and axial length measurements could not be obtained in all cases.

The anterior part of the GDD plate was positioned 6 mm from the limbus for all patients, with no need for plate trimming.

**Fig. 1:**
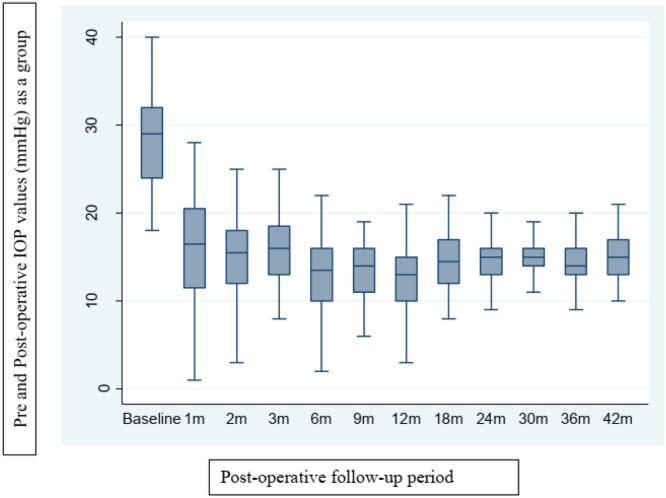
Box plot of baseline IOP variation and follow-up visits.

The 45-eyes had undergone an average of 3.62 ± 1.43 prior surgeries (range, 2–9), including trabeculotomies, goniotomies, trabeculectomies, needling, and cyclophotocoagulation. Four eyes had two prior surgeries, 24-eyes had three, 11 had four, three had six, two had seven, and one had nine previous procedures. The mean follow-up period after surgery was 37.78 ± 10.44 months (median, 42 months), with mean pre- and postoperative IOP of 28.18 ± 5.69 mmHg and 14.73 ± 2.90 mmHg, respectively (*p* < 0.05). The average number of hypotensive medications decreased from 2.96 ± 0.74 preoperatively to 1.40 ± 1.23 postoperatively (*p* < 0.05). (Table 2). Fig. 1 illustrates IOP trends across visits, showing the group's overall IOP pattern.

**Table 2:** Comparison of pre and postoperative values.

	Pre-operative	Post-operative
	*Average ± SD (median)*	*Average ± SD (median)*
**IOP**	28.18 ± 5.69 (29)	14.73 ± 2.90 (15)
**Number of hypotensive medications**	2.96 ± 0.74 (3)	1.40 ± 1.23 (1)

Eighteen eyes (40.0 %) from twelve children underwent initial postoperative assessments under sevoflurane anesthesia, transitioning to unsedated measurements as they reached the age of three during the study.

**Fig. 2:**
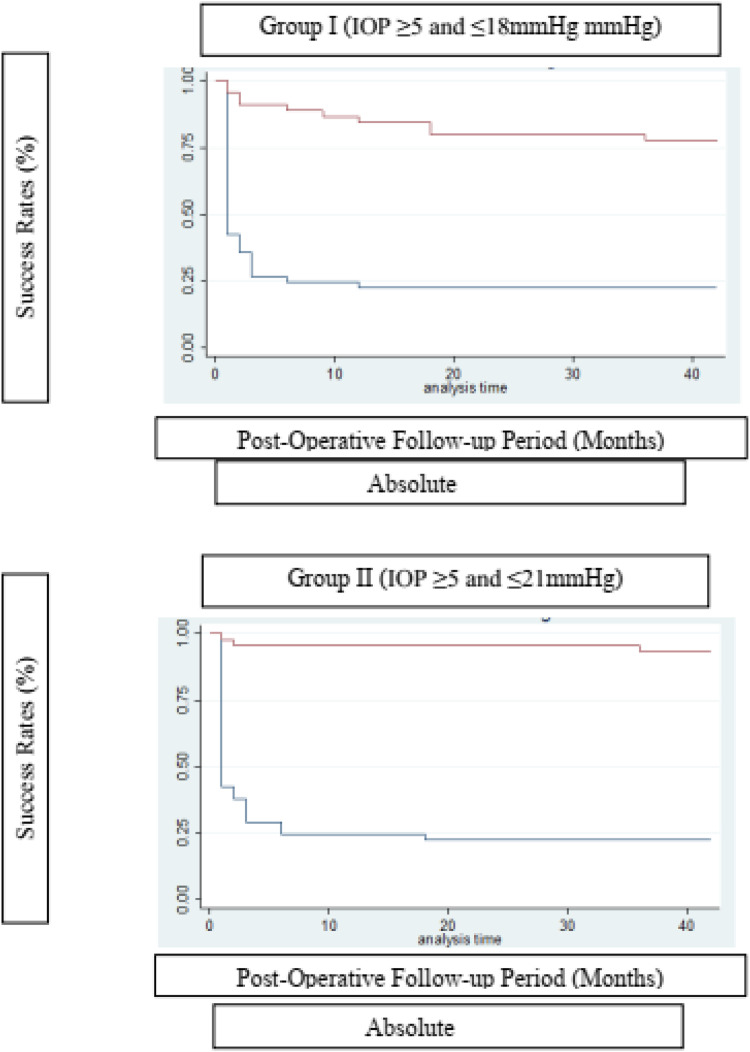
Kaplan-Meier Survival Estimates for both IOP ≤ 18 and IOP ≤ 21 mmHg, comparing Absolute and Qualified success. Implant survival curves for both criteria I and II, comparing Absolute and Qualified success.

Using absolute and qualified success criteria, the success rate of the Susanna UF® implant according to Criterion I ranged from 22.2 % (absolute success) to 93.3 % (qualified success) at the end of the 42-month follow-up period. For Criterion II, success rates ranged from 22.2 % (absolute success) to 77.8 % (qualified success) by the end of the same period. Fig. 2 presents the surgical survival curves.

The Log-rank test for equality of survival functions confirms a statistical difference between Absolute and Qualified success for both criteria of IOP ≤ 21 (*X* = 50.71; *p* < 0.0001) and IOP ≤ 18 mmHg (*X* = 34.56; *p* < 0.0001).

The survival rate of the Glaucoma Drainage Device (GDD) was notably higher in the qualified success group at the conclusion of the observation period (Table 3). To better delineate the IOP-lowering effect of the SUFGDD, patients were stratified by intraocular pressure levels (≥ 5 and < 12 mmHg, > 12 and 〈 15 mmHg, 〉 15 and 〈 18 mmHg, 〉 18 and 〈 21 mmHg, and 〉 21 mmHg), within both the absolute and qualified success groups for Criteria I and II (Table 4). The largest subset of patients was within the > 15 to < 18 mmHg range, using hypotensive eye drops (qualified success) for both Criteria I and II.

**Table 3:** SUFGDD survival analysis at different visits for different IOP criteria.

**Time (Months)**	**IOP ≥ 5 *E*≤21 mmHg**	**IOP ≥ 5 *E*≤21 mmHg**	**IOP ≥ 5 *E*≤18 mmHg**	**IOP ≥ 5 *E*≤18 mmHg**
	**Absolute**	**Qualified**	**Absolute**	**Qualified**
	*Average (%) ± SD*	*Average (%) ± SD*	*Average (%) ± SD*	*Average (%) ± SD*
3	37.8 (23.9 – 51.6)	97.8 (85.3 – 99.7)	35.6 (22.0 – 49.3)	93.3 (80.7 – 97.8)
6	24.4 (13.2 – 37.6)	95.6 (83.4 – 98.9)	24.4 (13.2 – 37.6)	88.9 (75.3 – 95.2)
9	24.4 (13.2 – 37.6)	95.6 (83.4 – 98.9)	24.4 (13.2 – 37.6)	86.7 (72.7 – 93.8)
12	24.4 (13.2 – 37.6)	95.6 (83.4 – 98.9)	22.2 (11.5 – 35.1)	84.4 (70.1 – 92.3)
18	22.2 (11.5 – 35.1)	95.6 (83.4 – 98.9)	22.2 (11.5 – 35.1)	80.0 (65.1 – 89.1)
36	22.2 (11.5 – 35.1)	93.3 (80.7 – 97.8)	22.2 (11.5 – 35.1)	77.8 (62.6 – 87.4)
42	22.2 (11.5 – 35.1)	93.3 (80.7 – 97.8)	22.2 (11.5 – 35.1)	77.8 (62.6 – 87.4)

**Table 4:** Distribution of final IOP in the absolute and qualified success groups, according to different levels of pressure reduction.

	IOP ≥ 5 *E* ≤ 21 mmHg	IOP ≥ 5 *E* ≤ 21 mmHg	IOP ≥ 5 *E* ≤ 18 mmHg	IOP ≥ 5 *E* ≤ 18 mmHg
	Absolute	Qualified	Absolute	Qualified
	n (%)	n (%)	n (%)	n (%)
**< 21 *e*>18 mmHg**	1 (2.22)	4 (8.88)	1 (2.22)	2 (4.44)
**< 18 *e*>15 mmHg**	1 (2.22)	17 (37.78)	1 (2.22)	14 (31.11)
**< 15 *e*>12 mmHg**	4 (8.88)	14 (31.11)	4 (8.88)	12 (26.67)
**< 12 *e*>5 mmHg**	4 (8.88)	7 (15.56)	4 (8.88)	7 (15.56)
**> 21 mmHg**	10 (22.22)	3 (6.67)	10 (22.22)	10 (22.22)
**Total**	45 (100.00)	45 (100.00)	45 (100.00)	45 (100.00)

To assess factors associated with surgical failure over time, cases with IOPs exceeding the upper success criteria limits (> 18 mmHg or > 21 mmHg) were analyzed. Factors assessed included prior surgeries, age at surgery, preoperative IOP, and number of preoperative eye drops (Table 5). In the qualified success, Criterion II group, complications significantly impacted the failure rate (HR = 6.50; 95 % CI 1.20–31.35; *p* = 0.029). For other criteria, there were no significant associations between failure and the number of prior surgeries, age at implantation, preoperative IOP, number of preoperative eye drops, or complications (*p* > 0.05).

**Table 5:** Evaluation of risk factors for surgical failure (Cox-Proportional Hazard Models).

	IOP ≥ 5 *E* ≤ 21 mmHg		IOP ≥ 5 *E* ≤ 21 mmHg		IOP ≥ 5 *E* ≤ 18 mmHg		IOP ≥ 5 *E* ≤ 18 mmHg	
	Absolute		Qualified		Absolute		Qualified	
	Hazard Ratio (95 % CI)	p-value	Hazard Ratio (95 % CI)	p-value	Hazard Ratio (95 % CI)	p-value	Hazard Ratio (95 % CI)	p-value
**Previous surgeries**	1.18 (0.95 – 1.46)	0.132	2.66 (0.89 – 7.94)	0.079	1.18 (0.95 – 1.47)	0.125	1.26 (0.87 – 1.81)	0.221
**Age**								
Younger than 2-years	Ref	‒	Ref	‒	Ref	‒	Ref	‒
2 to 6-years	0.84 (0.33 – 2.12)	0.717	0.19 (0.01 – 8.03)	0.416	0.83 (0.33 – 2.10)	0.702	1.89 (0.19 – 18.56)	0.586
6-years or more	1.45 (0.60 – 3.49)	0.411	0.45 (0.02 – 9.72)	0.608	1.40 (0.58 – 3.39)	0.449	5.10 (0.57 – 45.63)	0.145
**Pre IOP**	0.97 (0.91 – 1.04)	0.384	1.07 (0.82 – 1.38)	0.621	0.97 (0.91 – 1.04)	0.379	0.95 (0.85 – 1.07)	0.420
**Preop Number of drops**	1.43 (0.83 – 2.44)	0.194	0.06 (0.01 – 4.20)	0.195	1.43 (0.84 – 2.44)	0.190	1.30 (0.48 – 3.56)	0.608
**Complications**	1.40 (0.54 – 3.60)	0.490	10.40 (0.03 – 352.10)	0.118	1.43 (0.55 – 3.67)	0.459	6.50 (1.20 – 31.35)	0.029

Among the 45 operated eyes, complications occurred in seven cases (15.56 %) (Table 6), all of which required reoperation. Corneal touch was the most prevalent (6.67 %). Other complications were one cataract case, one implant exposure, and one hypotonia case and one endophthalmitis. Most complications (57 %) occurred within 1 to 6-months postoperatively. Firth's logistic regression was employed to examine factors linked to complications (Table 7), revealing no significant association between complications and any covariates, such as previous surgeries, age at surgery, preoperative IOP, or number of preoperative eye drops.

**Table 6:** Surgical complications and postoperative period in which they occurred.

	**n (%)**
**Complication**	
Tube-corneal touch	3 (6.67)
Cataracts	1 (2.23)
Endophthalmitis	1 (2.23)
Tube Exposure	1 (2.23)
Hypotony	1 (2.23)
**Postoperative Time**	
1 to 3 months	2 (28.57)
4 to 6 months	2 (28.57)
7 to 9 months	1 (14.29)
10 to 12 months	0 (0.00)
>12 months	2 (28.57)
**Needed reoperation**	
Yes	7 (100.00)
No	0 (0)

**Table 7:** Firth logistic regression for complication occurrence.

	Complication	
	Odds Ratio (95 % CI)	p-value
**Previous surgeries**	0.85 (0.42 – 1.72)	0.649
**Age**		
Younger than 2-years	Ref	‒
2 to 6-years	1.25 (0.20 – 7.70)	0.808
6 to 18-years	0.95 (0.13 – 6.79)	0.957
**Pre-Operative IOP**	1.04 (0.91 – 1.19)	0.569
**Number of hypotensive ocular drugs**	1.53 (0.47 – 4.94)	0.479

## Discussion

The management of PCG remains a challenge, due to intense inflammatory response, exacerbated scarring post-surgery, and the need for prolonged IOP control.^1^^,^^26^

When angled surgeries fail, trabeculectomies and GDDs have been widely used in both adult and pediatric patients. The reduced risks associated with the posteriorized bleb of GDDs, and the greater predictability in the immediate postoperative period, compared to trabeculectomies, have made GDDs preferable, mainly in children under 4-years-old. Implants have become an option for managing difficult or refractory cases, previously candidates only for cyclodestructive procedures.^9^, ^10^, ^11^, ^12^, ^13^^,^^27^

Several studies report on the use of drainage implants in pediatric patients, with success rates varying from 30 % to 90 % depending on the follow-up duration and type of glaucoma.^9^^,^^11^, ^12^, ^13^^,^^25^^,^^27^ Consensus indicates that implant success rates decline over time. For example, studies by Ou et al.^20^ and O'Malley Schotthoefer et al.^27^ reported cumulative success rates for Ahmed® implants of 63 % and 92 % at one year, decreasing to 33 % and 42 % by five and ten years, respectively. Rolim de Moura et al.^16^ reported a success rate of 58 % at 24-months, while van Overdam et al.^17^ found 44 % at 60 months. In Autrata et al.^28^ and Mandalos et al.,^29^ success rates at 6- and 8-years were 65 % and 40 %, respectively. All these studies used non-valved GDDs (Baerveldts® and Moltenos®).

In Brazil, the Susanna UF® implant (Kinner Silicone Rubber Indústria Comércio Ltda., Brazil) was released in 2017. To our knowledge, this is the first study reporting outcomes of the SUFGDD in a pediatric population. The authors hypothesized that this implant (Susanna UF®), being more flexible and thinner in comparison to similar devices, would yield promising results for childhood glaucoma.

Given the challenge of accurately measuring intraocular pressure in pediatric patients, obtaining axial length or corneal diameter measurements is important for PCG diagnosis. For the follow-up of children, axial length is more relevant than corneal diameter; however, its significance extends only up to three years of age. After this age range, the ocular globe no longer exhibits significant growth.Previous studies correlate corneal diameter with axial ocular length in congenital glaucoma.^30^^,^^31^ In the studied group, the average corneal diameter at surgery was 12.84 ± 0.75 mm, indicating that our population is severely buphthalmic.

While axial length measurements were not feasible for all cases, it was inferred that all eyes had lengths over 23.5 mm, reducing the likelihood of contact between the optic nerve and the posterior edge of the SUFGDD plate, as suggested by Freedman.^30^

Despite the severity of disease in our sample, results indicated effective IOP control over a follow-up period exceeding three years. The present series showed a qualified success rate of 95.6 % for Criterion I and 84.4 % for Criterion II at 12-months, slightly decreasing to 93.3 % and 77.8 %, respectively, at 36-months (Table 3).

Djodeyre et al.^12^ found that the number of prior glaucoma procedures and surgeon experience influenced implant survival, while Budenz et al.,^15^ Mandalos et al.^29^ and Morad et al.^10^ reported no significant associations between implant failure and factors such as previous surgeries, preoperative IOP levels, or number of preoperative glaucoma medications. In our series, surgeries were performed by a single experienced surgeon, and each patient had previously undergone two or more antiglaucoma procedures (including angle, filtering, and cyclodestructive surgeries), with a total ranging up to nine surgeries; the majority (53.3%) had undergone four procedures. No significant associations with failure were observed for prior surgeries (p > 0.05)

In the Criterion II group, surgical complications were significantly associated with failure (HR = 6.50; 95 % CI: 1.20–31.35; *p* = 0.029), suggesting that follow-up complications contributed to higher failure rates. In contrast to our findings, which showed no correlation between age and success rates, Munoz et al.^32^ reported an unfavorable influence of younger age on surgical success (Table 5). No significant associations with failure were observed for age at implantation, preoperative IOP, or the number of preoperative eye drops (*p* > 0.05). No significant differences were observed when stratifying by age groups (< 2-years, 2–6-years, and > 6-years) (Table 5).

In the series by Coleman et al.,^25^ Fellenbaum et al.^24^ and Englert et al.^9^ report that 50 %, 47 %, and 55.6 % of cases, respectively, required hypotensive medication postoperatively. In the present study, 75.6 % (34 of 45 eyes) required medication. The mean preoperative IOP was 28.18±5.69 mmHg, which decreased to 14.73±2.90 mmHg postoperatively. The mean number of eye drops decreased from 2.96±0.74 preoperatively to 1.40±1.23 postoperatively. Given that hypotensive drugs in pediatric patients are associated with systemic side effects, reducing their use is desirable.^1^ Chen et al.^11^ described a hypertensive phase in 25 % of cases (13 of 52 eyes). In this series, although all eyes received topical glaucoma medication from the first postoperative day, a distinct hypertensive phase was observed (Fig. 1). A significant reduction in IOP was documented at the two-month visit, followed by an increase at 18 months, at which time additional medications were necessary to maintain IOP stability up to 42 months. Early aqueous suppression has been associated with improved IOP reduction, higher success rates, and reduced hypertensive phase frequency.^33^

By the final follow-up, 11 of 45 eyes (22.2 %; 95 % CI: 11.5–35.1) achieved absolute success without antiglaucoma drugs according to either criterion (I and II).

Pediatric studies report high rates of complications such as hypotony, cataracts, retinal detachment, hemorrhagic choroidal detachment, and choroidal effusion, along with high reoperation rates and significant hypotensive medication use.^9^^,^^13^^,^^7^^,^^20^

In the present study, complications occurred in 7-eyes, giving a rate of 15.56 %. Six of these (85.71 %) retained vision, while one case led to irreversible vision loss due to endophthalmitis. No bilateral complications were observed.

Literature reports corneal endothelium touch as a common complication associated with drainage devices.^12^^,^^14^^,^^20^^,^^24^^,^^26^ A study on Ahmed® GDDs found approximately 73 % of intraocular implants migrated within five years,^34^ potentially causing intermittent endothelial trauma due to factors such as scleral elasticity, tissue remodeling, pressure changes, tube length, eye rubbing, and ocular growth.^14^^,^^20^^,^^24^

This complication was late-onset in 3 of the 45-eyes (6.67 %) and was successfully managed with tube repositioning and shortening. In a 6-year-old child, the touch was detected 5-months after implantation. In a 4-year-old, 9 m after. And in a 1-year-old, 15-months after.

Hypotony following tube opening is a common complication with non-valved GDDs, such as the Baerveldt®, occurring in up to 40 % of cases,^29^^,^^35^ but less frequent in valved implants.^9^^,^^25^

Djodeyre et al.^12^ documented a 25 % hypotony rate with Ahmed® GDDs. In this series, one eye developed hypotony (2.23 %) in a five-year-old patient, with an IOP of 3 mmHg at two months post-surgery. He had no history of cyclophotocoagulation but had undergone 4 previous surgeries (angular and fistulizing procedures). He maintained grade 3 athalamia, without choroidal peripheral detachment, for 2-months, without improvement. Tube restriction suture was performed at three months, with stable IOP achieved at 10 mmHg without medication by the final follow-up (40-months, Criterion II absolute success). Our low rates of hypotony is possibly a consequence of the narrow internal diameter of the SUFGDD.

A seven-year-old experienced conjunctival dehiscence and implant exposure after three prior surgeries. Conjunctival suturing with 10.0 nylon improved healing, achieving qualified success in Criterion II at 36-months. Exposure is a common cause of reoperation in both pediatric and adult populations.^36^^,^^37^ No cases of extrusion were observed. This may be attributed to the reduced anterior curvature (tube vault) of the SUFGDD when inserted into the anterior chamber.

One 10-year-old developed a cataract 15-months post-implantation. Phacoemulsification was successful. Cataract formation is often related to surgical manipulation, corticosteroid use, previous surgeries, and implant movement within the anterior chamber.^3^^,^^32^^,^^38^

One case of acute endophthalmitis, one month after surgery, led to vision loss in an eight-month-old with three prior surgeries. Although a vitrectomy was performed and the SUFGDD removed, the patient developed phthisis and blindness. No etiological agent was isolated in culture. No entry point for the pathogen was identified. The exact incidence of endophthalmitis in pediatric antiglaucoma surgeries is not known, but ranges from 0.8 % to 6.3 %, with an average of 2 %.^39^ In a large series of 542-eyes with Ahmed® implants, the rate of endophthalmitis for all ages, over a 9-year follow-up period, was found to be 1.7 %. Children had a five times higher rate of endophthalmitis compared to adults.^40^ Endophthalmitis can occur early or late after GDD surgery and is often associated with implant exposure (an important risk factor), and conjunctival discontinuities at points of erosion of the suture threads used to fix the plate.^40^

Complications in this study were evenly distributed over time, without indications of a learning curve effect.

The authors consider that the results of this study may help guide decision-making to preserve vision in this group of patients with PCG, a challenging vision threatening disease.

### Limitations

Study limitations include biases in IOP measurement (anesthesia, crying, corneal changes), impossibility of measuring ocular axial lenght in all children and population age heterogeneity. The advanced disease stage, the multiple prior surgeries, and the Brazilian tertiary care environment may limit generalizability. Also, the present results are based on procedures performed by a single surgeon.

Lastly, along the study, fourteen patients underwent SUFGDD surgery in both eyes, and fibrotic response could be similar bilaterally.

## Conclusion

The findings suggest that the SUFGDD is a safe and effective surgical option for refractory PCG cases.

## Declaration of competing interest

The authors declare no conflicts of interest.

## Appendix Supplementary materials

Image, application 1

Image, application 2
