# Supplementary material for: Outcomes of Susanna UF implants in refractory congenital glaucoma
Source: Clinics (Sao Paulo). 2025 May 1;80:100619. doi: 10.1016/j.clinsp.2025.100619 (PMC12090306; doi:10.1016/j.clinsp.2025.100619)
Supplement: Supplementary file 1 [file mmc1.docx]

**REFERENCES**

1. Ou Y, Caprioli J. Surgical management of pediatric glaucoma. Dev Ophthalmol. 2012;50:157–72.

2. Morales J, Al Shahwan S, Al Odhayb S, Al Jadaan I, Edward DP. Current surgical options for the management of pediatric glaucoma. J Ophthalmol. 2013;2013:763735.

3. Papadopoulos M, Edmunds B, Fenerty C, Khaw PT. Childhood glaucoma surgery in the 21st century. Eye Lond Engl. 2014;28(8):931–43.

4. Worst JG. GONIOTOMY; AN IMPROVED METHOD FOR CHAMBER-ANGLE SURGERY IN CONGENITAL GLAUCOMA. Am J Ophthalmol. 1964;57:185–200.

5. Mandal AK, Chakrabarti D. Update on congenital glaucoma. Indian J Ophthalmol. 2011;59 Suppl(Suppl1):S148-157.

6. Ikeda H. Long-term Outcome of Trabeculotomy for the Treatment of DevelopmentalGlaucoma. Arch Ophthalmol. 2004;122(8):1122.

7. Desai MA, Gedde SJ, Feuer WJ, Shi W, Chen PP, Parrish RK. Practice preferences for glaucoma surgery: a survey of the american glaucoma society in 2008. Ophthalmic Surg Lasers Imaging Off J Int Soc Imaging Eye. 2011;42(3).

8. Gedde SJ, Schiffman JC, Feuer WJ, Herndon LW, Brandt JD, Budenz DL, et al. Treatment outcomes in the Tube Versus Trabeculectomy (TVT) study after five years of follow-up. Am J Ophthalmol. 2012;153(5):789-803.e2.

9. Englert JA, Freedman SF, Cox TA. The Ahmed valve in refractory pediatric glaucoma. Am J Ophthalmol. 1999;127(1):34–42.

10. Morad Y, Donaldson CE, Kim YM, Abdolell M, Levin AV. The Ahmed drainage implant in the treatment of pediatric glaucoma. Am J Ophthalmol. 2003;135(6):821–9.

11. Chen A, Yu F, Law SK, Giaconi JA, Coleman AL, Caprioli J. Valved Glaucoma Drainage Devices in Pediatric Glaucoma: Retrospective Long-term Outcomes. JAMA Ophthalmol. 2015;133(9):1030–5.

12. Djodeyre MR, Peralta Calvo J, Abelairas Gomez J. Clinical evaluation and risk factors of time to failure of Ahmed Glaucoma Valve implant in pediatric patients. Ophthalmology. 2001;108(3):614–20.

13. Beck AD, Freedman S, Kammer J, Jin J. Aqueous shunt devices compared with trabeculectomy with Mitomycin-C for children in the first two years of life. Am J Ophthalmol. 2003;136(6):994–1000.

14. Donahue SP, Keech RV, Munden P, Scott WE. Baerveldt implant surgery in the treatment of advanced childhood glaucoma. J AAPOS Off Publ Am Assoc Pediatr Ophthalmol Strabismus. 1997;1(1):41–5.

15. Budenz DL, Gedde SJ, Brandt JD, Kira D, Feuer W, Larson E. Baerveldt glaucoma implant in the management of refractory childhood glaucomas. Ophthalmology. 2004;111(12):2204–10.

16. Rolim de Moura C, Fraser-Bell S, Stout A, Labree L, Nilfors M, Varma R. Experience with the Baerveldt glaucoma implant in the management of pediatric glaucoma. Am J Ophthalmol. 2005;139(5):847–54.

17. van Overdam KA, de Faber JTHN, Lemij HG, de Waard PWT. Baerveldt glaucoma implant in paediatric patients. Br J Ophthalmol. 2006;90(3):328–32.

18. Pakravan M, Esfandiari H, Yazdani S, Doozandeh A, Dastborhan Z, Gerami E, et al. Clinical outcomes of Ahmed glaucoma valve implantation in pediatric glaucoma. Eur J Ophthalmol. 2019;29(1):44–51.

19. Mofti A, Alharbi A, Alsuhaibani M, Aljaber A, Altamimi L, Ahmad S, et al. Long-term outcomes of the Ahmed glaucoma valve surgery in childhood glaucoma. J AAPOS Off Publ Am Assoc Pediatr Ophthalmol Strabismus. 2020;24(6):346.e1-346.e8.

20. Ou Y, Yu F, Law SK, Coleman AL, Caprioli J. Outcomes of Ahmed glaucoma valve implantation in children with primary congenital glaucoma. Arch Ophthalmol Chic Ill 1960. 2009;127(11):1436–41.

21. Freedman J. What is new after 40 years of glaucoma implants. J Glaucoma. 2010;19(8):504–8.

22. Biteli LG, Prata TS, Gracitelli CPB, Kanadani FN, Villas Boas F, Hatanaka M, et al. Evaluation of the Efficacy and Safety of the New Susanna Glaucoma Drainage Device in Refractory Glaucomas: Short-term Results. J Glaucoma. 2017;26(4):356–60.

23. Susanna FN, Susanna BN, Susanna CN, Nicolela MT, Susanna R. Efficacy and Safety of the Susanna Glaucoma Drainage Device After 1 Year of Follow-up. J Glaucoma. 2021;30(5):e231–6.

24. Fellenbaum PS, Sidoti PA, Heuer DK, Minckler DS, Baerveldt G, Lee PP. Experience with the Baerveldt implant in young patients with complicated glaucomas. J Glaucoma. 1995;4(2):91–7.

25. Coleman AL, Smyth RJ, Wilson MR, Tam M. Initial clinical experience with the Ahmed Glaucoma Valve implant in pediatric patients. Arch Ophthalmol Chic Ill 1960. 1997;115(2):186–91.

26. Jacobson A, Besirli CG, Bohnsack BL. Outcomes of Baerveldt Glaucoma Drainage Devices in Pediatric Eyes. J Glaucoma. 2022;31(6):468–77.

27. O’Malley Schotthoefer E, Yanovitch TL, Freedman SF. Aqueous drainage device surgery in refractory pediatric glaucomas: I. Long-term outcomes. J AAPOS Off Publ Am Assoc Pediatr Ophthalmol Strabismus. 2008;12(1):33–9.

28. Autrata R, Helmanova I, Oslejskova H, Vondracek P, Rehurek J. Glaucoma drainage implants in the treatment of refractory glaucoma in pediatric patients. Eur J Ophthalmol. 2007;17(6):928–37.

29. Mandalos A, Tailor R, Parmar T, Sung V. The Long-term Outcomes of Glaucoma Drainage Device in Pediatric Glaucoma. J Glaucoma. 2016;25(3):e189-195.

30. Margeta MA, Kuo AN, Proia AD, Freedman SF. Staying away from the optic nerve: a formula for modifying glaucoma drainage device surgery in pediatric and other small eyes. J AAPOS Off Publ Am Assoc Pediatr Ophthalmol Strabismus. 2017;21(1):39-43.e1.

31. Mendes MH, Sakata L, Betinjane AJ. [Central corneal thickness and its correlations with other ocular biometric data in patients with congenital glaucoma]. Arq Bras Oftalmol. 2011;74(2):85–7.

32. Munoz M, Tomey KF, Traverso C, Day SH, Senft SH. Clinical experience with the Molteno implant in advanced infantile glaucoma. J Pediatr Ophthalmol Strabismus. 1991;28(2):68–72.

33. Pakravan M, Rad SS, Yazdani S, Ghahari E, Yaseri M. Effect of early treatment with aqueous suppressants on Ahmed glaucoma valve implantation outcomes. Ophthalmology. 2014;121(9):1693–8.

34. Mathew DJ, Anuradha A, Low SAW, Belkin A, Buys YM, Trope GE. Long-term Follow-up of Ahmed Glaucoma Valve Tube Position Changes. J Glaucoma. 2019;28(3):276–80.

35. Stein JD, McCoy AN, Asrani S, Herndon LW, Lee PP, McKinnon SJ, et al. Surgical management of hypotony owing to overfiltration in eyes receiving glaucoma drainage devices. J Glaucoma. 2009;18(8):638–41.

36. Lai JS, Poon AS, Chua JK, Tham CC, Leung AT, Lam DS. Efficacy and safety of the Ahmed glaucoma valve implant in Chinese eyes with complicated glaucoma. Br J Ophthalmol. 2000;84(7):718–21.

37. Chen H, Zhang S xin, Liu L, Lin D, Tang X, Sun L, et al. [Intermediate-term and long-term clinical evaluation of the Ahmed glaucoma valve implantation]. Zhonghua Yan Ke Za Zhi Chin J Ophthalmol. 2005;41(9):796–802.

38. Rodrigues AM, Corpa MVN, Mello PA de A, de Moura CR. Results of the Susanna implant in patients with refractory primary congenital glaucoma. J AAPOS Off Publ Am Assoc Pediatr Ophthalmol Strabismus. 2004;8(6):576–9.

39. Results of the Endophthalmitis Vitrectomy Study. A randomized trial of immediate vitrectomy and of intravenous antibiotics for the treatment of postoperative bacterial endophthalmitis. Endophthalmitis Vitrectomy Study Group. Arch Ophthalmol Chic Ill 1960.1995;113(12):1479–96.

40. Al-Torbak AA, Al-Shahwan S, Al-Jadaan I, Al-Hommadi A, Edward DP. Endophthalmitis associated with the Ahmed glaucoma valve implant. Br J Ophthalmol. 2005;89(4):454–8.
