# Supplementary material for: Outcomes of Susanna UF implants in refractory congenital glaucoma
Source: Clinics (Sao Paulo). 2025 May 1;80:100619. doi: 10.1016/j.clinsp.2025.100619 (PMC12090306; doi:10.1016/j.clinsp.2025.100619)
Supplement: Supplementary file 2 [file mmc2.docx]

**Orcid and Author contribution**

Aline Domingos Pinto Ruppert

ORCID 0000-0001-6028-4031

Conceptualization, Data curation, Writing Original draft, Review & Editing project administration

Nara Gravina Ogata

ORCID [0000-0003-3019-8102](https://orcid.org/0000-0003-3019-8102)

Methodology

Leopoldo Ernesto Oiticica Barbosa

ORCID [0000-0002-6112-8409](https://orcid.org/0000-0002-6112-8409)

Resources and Visualization

Paulo Silas Neroni Stina

ORCID [0009-0004-7445-8740](https://orcid.org/0009-0004-7445-8740)

Software

Marcus Vinícius Takatsu

ORCID [0000-0003-0034-4455](https://orcid.org/0000-0003-0034-4455)

Methodology, Software

Ernst Werner Oltrogge

ORCID [0009-0003-9255-2426](https://orcid.org/0009-0003-9255-2426)

Validation and Investigation

Marcelo Hatanaka

ORCID [0000-0002-0244-3652](https://orcid.org/0000-0002-0244-3652)

Reviewing, Editing and supervision
